# Risk of low birth weight on adulthood hypertension - evidence from a tertiary care hospital in a South Asian country, Sri Lanka: a retrospective cohort study

**DOI:** 10.1186/s12889-017-4268-x

**Published:** 2017-04-24

**Authors:** Dileepa Senajith Ediriweera, Nuwani Dilina, Usha Perera, Francisco Flores, S. Samita

**Affiliations:** 1 Centre for Health Informatics, Biostatistics and Epidemiology, Faculty of Medicine, University of Kelaniya, Ragama, Sri Lanka; 2 Ministry of Health, Colombo, Sri Lanka; 3 Japan International Corporation Agency, Evidence Based Management Study, Colombo, Sri Lanka; 4 Postgraduate Institute of Agriculture, University of Peradeniya, Peradeniya, Sri Lanka

**Keywords:** Birth weight, Adult blood pressure, Linear logistic model, Risk factors, Adjusted effect

## Abstract

**Background:**

Although low birth weight (LBW) is common in South Asian region there are not many studies being done to evaluate LBW and adulthood hypertension association in this region, including in Sri Lanka. Although this association has been studied in other regions, most studies have not evaluated this association in the presence of socioeconomic and lifestyle factors. This study was conducted to investigate whether low birth weight (LBW) is associated with adulthood hypertension after adjusting for other potential risk factors of hypertension.

**Methods:**

Nearly 15,000 individuals born during 1950 to 1965 were selected and invitations were sent to their original addresses. Out of them 217 individuals responded and among them birth weight was recovered for 122 individuals. Separate linear logistic models were fitted to model high systolic blood pressure (SBP: systolic blood pressure > 140 mmHg), high diastolic blood pressure (DBP: diastolic blood pressure > 90 mmHg) and hypertension (either SBP > 140 mmHg or DBP > 90 mmHg).

**Results:**

Separate linear logistic model fitting revealed LBW having a significant association with high SBP (OR = 2.89; 95% CI: 1.01 to 8.25; *P* = 0.04), and hypertension (OR = 3.15; 95% CI: 1.17 to 9.35; *P* = 0.03), but not with high DBP (OR = 0.75; 95% CI: 0.22 to 2.16; *P* = 0.62), when effect of LBW was studied after adjusting for all other potential risk factors.

**Conclusions:**

LBW has a tendency to cause high adult blood pressure in South Asian region, and the findings are consistent with previous work on LBW and adulthood hypertension association in other regions of the world.

## Background

Hypertension is considered as the leading global burden of disease risk factor [1]. Globally, incidence of hypertension is on the rise and the prevalence of high blood pressure has been estimated as 22%. Hypertension causes 7.5 million deaths worldwide, which represent about 12.8% of total all deaths and high blood pressure is identified as a major risk factor for coronary heart diseases and hemorrhagic strokes [2, 3].

Baker et al. [4, 5] have demonstrated fetal low birth weight can lead to raised blood pressure in later life and many studies done in different geographical locations, systematic reviews and meta-analysis have shown an inverse relationship between birth weight and adult blood pressure [6–11].

However, one of the major criticism for this is, in developing countries, infant weight is often considerably lower than it is in developed countries and yet cardiovascular disease is less prevalent [12, 13]. The highest proportion of low birth weight babies in the world are born in South Asian region [14] and there is little evidence to demonstrate birth weight blood pressure relationship from this region including Sri Lanka where low birth weight babies are born 16.7 per 100 births [15] and yet no studies have been done until now. Even in other regions, although studies have investigated the birth weight effect along with the age, gender and anthropometric measurements of individuals on adult blood pressure [16, 17], many have failed to investigate the effect of lifestyle, on birth weight and adult blood pressure relationship [18].

This study was conducted to determine whether there is an association between birth weight and adult blood pressure in Sri Lanka as a south Asian country where the occurrence of cardiovascular disease is relatively low and if any, to investigate whether this association is consistent with that in developed countries. The study was based on a tertiary care hospital in Sri Lanka and the association between birth weight and adult blood pressure was investigated after adjusting for the other risk factors of hypertension [9].

## Methods

The study, a retrospective cohort study, was conducted at the Castle street hospital for women in 2007. The Castle street hospital is the first hospital to record birth weight in Sri Lanka in 1950 and the hospital is considered as one of the largest maternal tertiary care hospitals in Sri Lanka. All the live births from 1950 to 1966 were selected for the study. Hospital’s paper based birth registries contained offspring’s date of birth, gender, birth weight and parents’ names and postal addresses. Nearly 15,000 invitations were sent to the original addresses of the parents, explaining the study purposes and requested to hand over the invitation or to convey the message if contactable, to the person who was born to the named person on a particular date at the Castle street hospital for women. A prepaid postcard was attached along with the invitation and offspring was requested to fill the current contact information and post it back to the research office without any additional expense. A total of 217 responded to the invitations and among them birth weight were recovered from 122 individuals.

Ethical clearance for the study was obtained from the Ethics Review Committee of Sri Lanka Medical Association. Informed written consent was obtained from each subject before participating to the study. Follow up forum was arranged to deliver the blood investigation results, explain the individual risk factors for non–communicable diseases (NCDs) as well as to educate on diet, exercise, stress management and, controlling smoking and alcohol habits to prevent NCDs.

The study was conducted according to guidelines of World Health Organization STEPwise approach to surveillance of NCDs [19]. The questionnaire had three components; namely i) an interviewer-administered questionnaire evaluating socio-demographic characteristics and lifestyle, family history of diseases, past medical history, ii) physical examination of blood pressure, height, weight, waist circumference, hip circumference and iii) blood investigations of lipid profile and fasting glucose levels. Lifestyle assessment included evaluating smoking habits, alcohol consumption, physical activity, fruit consumption, vegetable consumption and rice consumption.

Initially the interviewer administered questionnaire were given and later physical examination and blood investigations were done. Blood Pressure was recorded in participants after 15 min of sitting by using an OMRON IA2 digital automatic blood pressure monitor. Blood pressure was measured on the left arm using the appropriate cuff size placed at the level of heart, where arm was rested on a table with the palm facing upwards. Three blood pressure recordings were obtained 3 min apart and mean blood pressure of the last two readings were considered for the analysis. Height measuring board was used to measure the height, where participants were asked to remove foot wear, head gear and keep the feet together, heels against the back board and knees straight with eyes at the same level as ears. A calibrated electronic portable weighing scale was used to measure the participants weight with stiff wooden board placed under the scale. Separate private room was arranged to measure the waist and hip circumference and measurements were done using a constant tension tape. Waist circumference was measured directly over the skin at the midpoint of the lower palpable margin of the rib and top of the iliac crest, this was done at the end of a normal expiration with arms relaxed either side. Hip circumference was measured directly over the skin at the maximum circumference over the buttocks with arms relaxed on either sides. All the interviews and examinations were done by qualified doctors after providing a training session.

Study participants were categorized into two groups based on their birth weight; low birth weight (LBW) i.e. birth weight less than 2.5 kg and normal birth weight (NBW) otherwise. Note that there was a single participant with birth weight more than 4 kg (i.e. birth weight of 4.03 kg) and was categorize to NBW group. Group comparisons (i.e. NBW vs LBW) were done using Student *t* test, Wilcoxon Rank Sum test Pearson’s chi-square test and Fisher’s exact test as appropriate. Less fruit intake was defined as consumption of less than 2 servings per day, less vegetable intake was defined as consumption of less than 3 servings per day and more rice consumption was defined as consumption of more than 3 rice cups per day.

Separate linear logistic models were fitted for systolic blood pressure (SBP), diastolic blood pressure (DBP) and hypertension. High SBP was defined as SBP >140 mmHg irrespective of DBP and similarly the definition for high DBP was DBP >90 mmHg irrespective of SBP. Overall hypertension was defined as either SBP > 140 mmHg or DBP >90 mmHg. Initially, exposure variables were screened for model fitting using Pearson’s Chi-square test and simple linear logistic regression, for nominal and continuous variables respectively, and the significant variables at *P* = 0.2 level were then used for model fitting. Since the data are binary, Hosmer and Lemeshow goodness of fit test [20] was used to evaluate the adequacy of the fitted models. Effect of each exposure variable was studied after adjusting for other variables in the linear logistic model (type III effect) using the change in the Deviance, ΔG^2^ [21]. The difference between the levels of the exposure variables was evaluated using odds ratios, confident interval of the odds ratio and *P* value. Analysis were performed using SAS University Edition and R Programming Language version 3.2.5.

## Results

Of 122 subjects, there were 72 males (59%) and 50 females (41%) with mean age (standard deviation; SD) of 51 (3.9) years. The mean birth weight (SD) of the subjects were 2.8 (0.4) and 26 out of 122 (21.3%) had LBW. Distribution of sample characteristics of LBW and NBW groups are shown in Table 1.

**Table 1:** Distribution of sample characteristics among low birth weight (LBW) and normal birth weight (NBW) group

Variable	LBW (*n* = 26)	NBW (*n* = 96)	*P* value
Males (%), number	12 (46.1)	38 (39.6)	0.7
Age (SD), years	49 (10.7)	51 (3.9)	0.5
Weight (SD), kg	61 (9.7)	65 (12.4)	0.8
Height (SD), cm	155 (13.2)	158 (8.3)	0.2
Hip (SD), cm	96 (8.6)	98 (9.9)	0.4
Waist (SD), cm	91 (7.1)	91 (10.4)	0.9
BMI (SD), kg/m^2^	25.83 (3.35)	25.91 (4.57)	0.9
Smokers (%), number	2 (7.7)	14 (14.7)	0.5
Alcohol consumers (%), number	8 (33.3)	20 (23.0)	0.4
Major ethnicity (%), number	24 (92.3)	93 (96.9)	0.3
Married (%), number	24 (96.0)	82 (94.2)	1.0
Income (%), number			
< Rs. 5000	3 (11.5)	10 (10.4)	0.6
Rs. 5001 – Rs.10,000	9 (34.6)	26 (27.1)	
Rs. 10,001 – Rs.20,000	10 (38.5)	29 (30.2)	
>Rs. 20,001	4 (15.4)	31 (32.3)	

Initial variable screening showed, high SBP associated with age, less fruit consumption and LBW; high DBP associated with age, smoking, alcohol consumption, less fruit consumption, less vegetable consumption, height, family history of hypertension, diabetes and hypercholesterolaemia; and hypertension associated with age, LBW, height, family history of myocardial infarction, hypertension and cancer (Table 2).

**Table 2:** *P* values of initial variable screening prior to model fitting

Study variables	Response variable		
	High SBP	High DBP	hypertension
Age	0.01	0.14	< 0.01
Gender	0.90	0.87	0.50
Ethnicity	0.97	0.95	0.70
Marital Status	0.99	0.99	0.95
^a^Income	0.29	0.63	0.69
^a^Smoking	0.46	0.14	0.22
^a^Alcohol consumption	0.53	0.14	0.41
Family History of Cardiovascular accidents	0.79	0.54	0.66
Family History of Myocardial Infarction	0.38	0.52	0.07
Family History of Hypertension	0.66	0.05	0.05
Family History of Diabetes Mellitus	0.70	0.09	0.29
Family History of Hypercholesterolaemia	0.78	0.15	0.98
Family History of Cancer	- ^b^	0.38	0.19
^a^More rice consumption	0.73	0.59	0.93
^a^Less fruit consumption	0.06	0.02	0.34
^a^Less vegetable consumption	0.98	0.12	0.63
Low Birth Weight	0.09	0.77	0.05
^a^Weight	0.51	0.49	0.50
^a^Body mass index	0.69	0.06	0.54
^a^Hip circumference	0.43	0.64	0.93
^a^Waist circumference	0.87	0.23	0.42

*SBP* Systolic Blood Pressure, *DBP* Diastolic Blood Pressure

^a^Values are at the time of data collection, ^b^No cases available for testing

When the linear logistic models were fitted using all selected exposure variables from screening, the best fitting models were as follows.

For High SBP;

$$ logit\ \left( High\  SBP\right)=-9.07+1.06 LBW+1.02 Less\  Fruits+0.15 Age $$

For High DBP;

$$ logit\ \left( High\  DBP\right)=-1.85+0.94 family\  history\  of\  HTN $$

For hypertension;

$$ logit\ (hypertension)=-11.25+1.15 LBW+0.21 Age $$

Hosmer and Lemeshow Goodness of fit test showed, fitted models for high SBP (*P* = 0.41), high DBP (*P* = 1.00) and hypertension (*P* = 0.60) were adequate.

According to best fitting models High SBP is associated with LBW, age and less fruit consumption, whereas High DPB is associated with family history of hypertension and hypertension is associated with age and LBW. Thus the risk factors for high SBP are LBW (*P* = 0.04), advancing age (*P* = 0.01) and less fruit intake (*P* = 0.02). On the other hand, only family history of hypertension (*P* = 0.04) showed a significant effect on high DBP. LBW was found to be a risk factors of hypertension too (*P* = 0.03). In addition, advancing age was also found to be a risk factor for hypertension (*P* < 0.01). Estimated odds ratios, 95% confident interval of the odds ratio and *P* value of the risk factors of above three models are given in Table 3.

**Table 3:** Association between birth weight and blood pressure

	OR (95% CI)	*P*
Systolic Blood Pressure		
Low birth weight	2.89 (1.07 to 8.25)	0.04
Age	1.16 (1.04 to 1.32)	0.01
Less fruit consumption	2.78 (1.21 to 6.71)	0.02
Diastolic Blood Pressure		
Low birth weight	0.75 (0.22 to 2.16)	0.62
Family history hypertension	2.61 (1.06 to 6.94)	0.04
Hypertension		
Low birth weight	3.15 (1.17 to 9.35)	0.03
Age	1.24 (1.11 to 1.41)	<0.01

*P* – Type 3 significance level

## Discussion

In this study it was revealed that low birth weight is a primary risk factor for adult hypertension and the findings of this study are consistent with inverse relationship of birth weight and adult hypertension in a South Asian country as well as previously shown in other geographical locations including in both developing and developed countries [8, 13, 22].

Low birth weight showed a significant effect on hypertension specifically with high SBP in adulthood, but not directly with high DBP. Interestingly when both SBP and DBP was considered to define hypertension, the effect of LBW was much more significant compared to the individual effect on SBP and DBP, demonstrating the cumulative effect of LBW on both SBP and DBP on determining adult hypertension.

Age appeared as an independent risk factor for high SBP and hypertension, and this emphasizes the importance of regular blood pressure screening in all individuals with the advancement of age, irrespective of their birth weight. Similarly, less fruit intake and family history of hypertension are the risk factors for SBP and DBP respectively, indicating the importance of consuming more fruits to minimize the risk of hypertension and the regular screening of blood pressure, especially in those who have a family history of hypertension.

The south Asian region is considered as the region giving birth to the highest proportion of low birthright babies in the world [14] and the findings of the study are important as Sri Lanka being one of the countries from this region. Since LBW babies are more prone to develop hypertension in adulthood, and demands health care facilities imposing an economic burden to developing countries like Sri Lanka. This study provides an important message to health care decision makers, to improve antenatal care and screening facilities to identify the probable low birth weight infants prior to the delivery stage and to take necessary measures to minimize the effect of such causative agents on birth weight. Once such low birth weight delivery is encountered it is important to follow up such individuals and screening them for hypertension and to adopt healthy lifestyle practices.

There were few limitations in this study. Though we sent 15,000 invitations to the original postal address of the parents we could track down only 217 subjects born during 1950 to 1966 at the Castle street hospital for women, Sri Lanka possibly because the subjects have left their original birthplaces or changes have taken place in the mailing addresses and the low response rate is unlikely to affect by the low literacy rate of the dwellers of the original postal addresses as Sri Lanka has a literacy rate of 91% according to the Sri Lanka Labour Force Survey 2007. Out of the 217 subjects, birth weights were recovered only from the 122 due to the destruction of the particular pages of the manually maintained records. Even though local authorities had understood the importance of recording birth weight in early as 1950, proper record maintenance was not adopted which in turn indicates the requirement of electronic record keeping systems in developing countries like in Sri Lanka. As a result, our study has been based on a small sample size, and, consequently, the risk estimates included wide confidence intervals. Nevertheless, the magnitude of the risk of hypertension associated with low birth weight was substantially strong in this population, and the risk estimate remained statistically significant.

Also we could not study the potential perinatal factors which can influence birth weight of a child, including gestational age, parity, maternal smoking, socio-economic status of parents, as such information was not available in birth registries. Prevalence of maternal smoking is very low in Sri Lanka and hence effect on the study can be considered as negligible. Even though, Castle street hospital for women is a tertiary care hospital, it is not only serving for high risk pregnancies as free Sri Lankan government healthcare service allows general public to select their health facility on individual preference, therefore study is unlikely to include majority of high risk pregnancies. Since only 25% were home deliveries in 1950s in Sri Lanka [23], we can consider home delivery rate may not have effect on the results of the study. When defining variables, such as income, smoking, weight, physical activity as well as alcohol, rice, vegetable and fruit consumption, values at the time of data collection was recorded. This might lead to dilute the evidence for the association. For instance, suppose a subject was found to consume more rice and this subject has started consuming more rice quite recently. Then it is highly unlikely that the effect of high rice consumption is reflected in the response variable due to the fact that often there is a time lag between exposure and developing a disease. Thus, it would have been better if the data was available for exposure variables of the form more rice consumed years instead of consume more rice or not at the time of data recording.

Although the study findings may not be generalized to the region, this study provides important evidence for the association between LBW and adult hypertension from the region and authors encourage other countries to investigate birth weight and adult hypertension association.

## Conclusions

In conclusion, we have shown LBW has a significant effect on hypertension in adulthood specifically with respect to high SBP after adjusting for other risk factors of hypertension and the present study supports the consistency of LBW and adult hypertension association in other regions of the world.

## Abbreviations

DBP: Diastolic blood pressure
G^2^: Deviance
LBW: Low birth weight
NBW: Normal birth weight
SBP: Systolic blood pressure
SD: Standard deviation
